# Deep Sequencing of Human Nuclear and Cytoplasmic Small RNAs Reveals an Unexpectedly Complex Subcellular Distribution of miRNAs and tRNA 3′ Trailers

**DOI:** 10.1371/journal.pone.0010563

**Published:** 2010-05-14

**Authors:** Jian-You Liao, Li-Ming Ma, Yan-Hua Guo, Yu-Chan Zhang, Hui Zhou, Peng Shao, Yue-Qin Chen, Liang-Hu Qu

**Affiliations:** State Key Laboratory of Biocontrol, Key Laboratory of Gene Engineering of the Ministry of Education, Sun Yat-sen University, Guangzhou, People's Republic of China; New England Biolabs, Inc, United States of America

## Abstract

**Background:**

MicroRNAs (miRNAs) are ∼22-nt small non-coding regulatory RNAs that have generally been considered to regulate gene expression at the post-transcriptional level in the cytoplasm. However, recent studies have reported that some miRNAs localize to and function in the nucleus.

**Methodology/Principal Findings:**

To determine the number of miRNAs localized to the nucleus, we systematically investigated the subcellular distribution of small RNAs (sRNAs) by independent deep sequencing sequenced of the nuclear and cytoplasmic pools of 18- to 30-nucleotide sRNAs from human cells. We identified 339 nuclear and 324 cytoplasmic known miRNAs, 300 of which overlap, suggesting that the majority of miRNAs are imported into the nucleus. With the exception of a few miRNAs evidently enriched in the nuclear pool, such as the mir-29b, the ratio of miRNA abundances in the nuclear fraction versus in the cytoplasmic fraction vary to some extent. Moreover, our results revealed that a large number of tRNA 3′trailers are exported from the nucleus and accumulate in the cytoplasm. These tRNA 3′ trailers accumulate in a variety of cell types, implying that the biogenesis of tRNA 3′ trailers is conserved and that they have a potential functional role in vertebrate cells.

**Conclusion/Significance:**

Our results provide the first comprehensive view of the subcellular distribution of diverse sRNAs and new insights into the roles of miRNAs and tRNA 3′ trailers in the cell.

## Introduction

MicroRNAs (miRNAs) are ∼22-nt long non-coding regulatory RNAs that are widely expressed in metazoans and regulate many important biological processes, including differentiation, apoptosis and cellular transformation [1]. Most miRNA genes are transcribed by RNA polymerase II into primary miRNA transcripts, which are further processed into hairpin-structured miRNA precursors (pre-miRNAs) in the nucleus by Drosha and its partner DGCR8/Pasha [2]. Pre-miRNAs are then exported to the cytoplasm by Exportin5 [3] and converted into ∼22-nt mature miRNAs by Dicer, after which one strand of the newly formed duplex is incorporated into the Ago protein complex [4], [5]. miRNAs are generally believed to inhibit mRNA translation post-transcriptionally by binding partially complementary target sites in the 3′ untranslated regions (UTRs) of target mRNAs in the cytoplasm [6]. However, recent studies have shown that some miRNAs are localized to the nucleus. For instance, several rat miRNAs localize to the nucleolus [7], [8] and human miR-29b contains a nuclear import element at its 3′ end that can direct nuclear enrichment of this miRNA [9]. Moreover, miRNAs can inhibit or activate gene expression at the transcriptional level in the nucleus of human and plant cells [10]–[12]. Although only a few miRNAs have been identified in the nucleus thus far, it is very likely that many more miRNAs localize to and function in the nucleus. Identification of nuclear miRNAs may provide new insights into the regulatory roles played by miRNAs in the nucleus.

Humans express four Ago proteins (Ago1-Ago4) in numerous tissues and cell types [13]. All four of these Ago proteins associate with miRNAs and other small RNAs (sRNAs) [14]–[16] and they all contribute to the process of miRNA-mediated gene silencing [17]. The Ago2 protein, a key component of the RISC complex, can be imported into the nucleus from the cytoplasm [18], [19]. To date, the exact nuclear function of the Ago2 protein remains unclear but treatment with exogenous siRNAs (which associate with the Ago2 protein after the introduction of double-stranded RNAs (dsRNAs) into the cell) directed against nuclear RNAs such as the 7SK RNA efficiently reduce 7SK RNA levels in the nuclear fraction [20]. These findings suggest that the Ago2 protein can mediate the cleavage of target RNAs in the nucleus. The Ago1 protein is also known to localize to and function in the nucleus [21], [22]. It is unclear whether the Ago proteins can enter the nucleus while bound to cytoplasmic sRNAs. Notably, the nuclear localization of NRDE-3, an Ago protein of *Caenorhabditis elegans*, requires sRNA binding [23].

Recently, deep sequencing of 18- to 30-nt fractionated RNA has become the most common and widely used approach for the discovery of sRNAs. This is a transcriptome-wide approach and is highly effective even for the identification of very low-abundance sRNAs. However, this approach does not provide information on the subcellular localization of the sRNAs. This additional information is often very useful in characterizing the functions of the sRNAs. In fact, many small regulatory RNAs, including endogenous siRNAs in worms [23], heterochromatin-related siRNAs in fungi [24] and piRNAs (piwi-associated RNAs) in mammals [25], are believed to function at least partially within the nucleus. In contrast, miRNAs are generally believed to function predominantly in the cytoplasm based on their subcellular localization. Next-generation sequencing technology has been used extensively for the discovery and profiling of miRNAs in cells [26]–[28]. This technology could also provide a powerful approach for investigating the subcellular localization and function of sRNAs by combining it with subcellular fractionation techniques. In this study, we performed a deep sequencing analysis of sRNAs isolated from both the cytoplasm and the nucleus of human cells. Our results provide the first evidence of an unexpectedly complex subcellular distribution of diverse sRNAs. Specifically, we provide a comprehensive examination of the patterns of distribution of miRNAs and tRNA 3′ trailers in human cells.

## Results

### Isolation of nuclear and cytoplasmic RNAs

**Figure 1:**
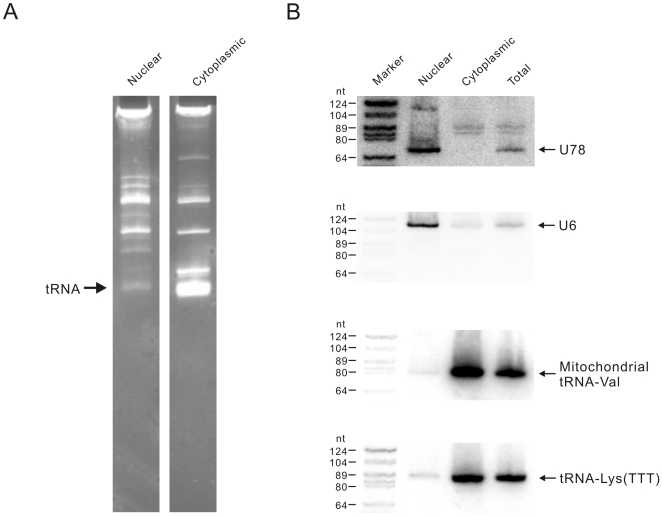
Quality evaluation of nuclear and cytoplasmic RNAs. (*A*) Quality analysis of nuclear and cytoplasmic RNAs was performed using 10% denaturing polyacrylamide gels. Approximately 15 micrograms (µg) of nuclear and cytoplasmic RNA was loaded on 10% denaturing polyacrylamide gels and stained using ethidium bromide. (*B*) Northern blot assays were conducted to evaluate the purity of the nuclear and cytoplasmic RNAs. Equal amounts (25 µg) of nuclear, cytoplasmic and total RNA were used to detect the U78 snoRNA, U6, the mitochondrial tRNA-Val, and the tRNA-Lys (TTT).

Nuclear and cytoplasmic RNAs were isolated from the human nasopharyngeal carcinoma (NPC) 5-8F cell line, which was derived from the SUNE-1 cell line [29]. The efficiency of the nuclear/cytoplasmic fractionation was assessed by electrophoresis of RNA on 10% denaturing polyacrylamide gels (Figure 1A). tRNAs, which are located predominantly in the cytoplasm [30], were almost entirely depleted in the nuclear RNA fraction and abundant in the cytoplasmic fraction, indicating that nuclear and cytoplasmic RNAs were successfully separated. Northern blot analysis of three different compartment-specific RNAs was further used to evaluate the purity of the nuclear and cytoplasmic RNA fractions (Figure 1B). The U78 snoRNA (small nucleolar RNAs) and U6 snRNA were present exclusively in the nuclear RNA fraction, whereas the nuclear-encoded tRNA and mitochondrial-encoded tRNA were enriched in the cytoplasmic RNA. Together, these results indicated that the nuclear and cytoplasmic RNAs were highly purified. Northern blot analysis of U78 snoRNA and tRNA-Lys (TTT) indicated that the proportions of nuclear RNA in the 5-8F cell total RNA were ∼20%, the value that is in agreement with that estimated previously for HeLa cells [16].

### Profiling of sRNAs in the nucleus and the cytoplasm

**Figure 2:**
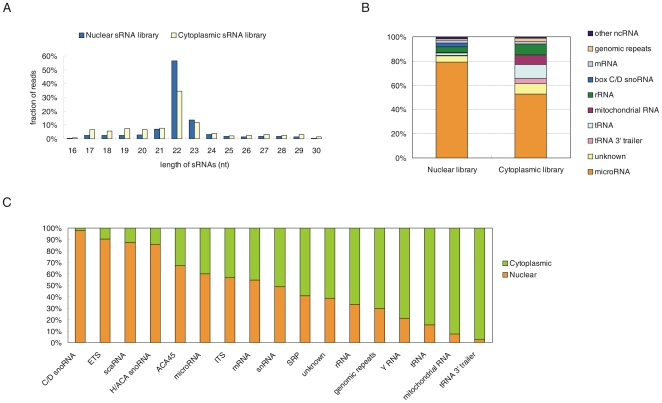
Nuclear and cytoplasmic sRNA libraries. (*A*) Length distribution of nuclear and cytoplasmic sRNAs. (*B*) Composition of nuclear and cytoplasmic sRNA libraries. (*C*) Comparison of the abundance of each sRNA class in the nuclear and cytoplasmic sRNA libraries, respectively. To show the difference more clearly, we combined all sequences from our two libraries after the read count of each sequence was normalized to RPM (reads per million), then calculated the fraction of the different kinds of sRNAs in the nuclear and cytoplasmic sRNA libraries. ACA45 scaRNA-derived sRNAs are excluded from the other scaRNA-derived sRNAs in the schematic. Nuclear indicates nuclear sRNA library, cytoplasmic indicates cytoplasmic sRNA library.

The 18- to 30-nt sRNAs isolated from the nuclear and cytoplasmic RNA fractions were sequenced using the Illumina high-throughput sequencing platform. The data were filtered by removing those reads without a 3′ adaptor sequence, 5′ adaptor contaminants, poly-A reads, reads with size less than 16-nt and reads that did not map to the human genome (see Methods). After filtering, the nuclear and cytoplasmic sRNA libraries contained 5,321,867 and 4,079,549 reads, corresponding to 337,547 and 236,122 unique sequences, respectively. Remarkably, the length distribution of the nuclear sRNAs was similar to the length distribution of the cytoplasmic sRNAs, with a peak at 22-nt (Figure 2A).

The sRNA sequences were annotated according to their overlap with sequences of known RNAs. The annotation results demonstrated that the nuclear and cytoplasmic sRNA libraries contained the same classes of sRNAs (Table 1, Figure 2B). However, the abundance of some classes differed significantly between the two libraries (Figure 2C). The five most abundant sRNA classes in the nuclear sRNA library were miRNAs and sRNAs derived from rRNAs, snoRNAs, mRNAs and tRNAs. In contrast, the five most abundant sRNA classes in the cytoplasmic sRNA library were miRNAs and sRNAs derived from tRNAs, rRNAs, mitochondrial RNAs and tRNA 3′ trailers. Both libraries also contained small portions of sRNAs that mapped to un-annotated genomic regions (5.3% in the nuclear sRNA library, 8.4% in the cytoplasmic sRNA library).

**Table 1:** Raw read counts (non-normalized) of diverse sRNA classes.

	Nuclear sRNA library	Cytoplasmic sRNA library
total	5321867	4079549
box C/D snoRNA	174833	3132
external transcribed spacer of rRNA	40913	3266
box H/ACA snoRNA	2780	353
scaRNA	14027	5175
miRNA	4208498	2156495
internal transcribed spacer of rRNA	3225	1879
mRNA	117038	74840
snRNA	13881	11140
SRP RNA	2919	3256
rRNA	237973	367524
Y RNA	8033	22629
tRNA	111904	476974
mitochondrial RNA	33690	311742
tRNA 3′ trailer	6295	180657
genomic repeat	64527	117314
miscRNA	1119	1281
unknown	280215	341913

miscRNA includes 7SK RNA, vRNA, RNaseMRP RNA, RNaseP RNA, Xist, H19.

As expected, the most abundant class of sRNAs (52.9%) in the cytoplasmic sRNA library was the class of known miRNAs. Strikingly, a higher proportion of miRNAs (79.1%) was observed in the nuclear sRNA library. The abundance of other sRNAs in both the nucleus and the cytoplasm generally correlated with the abundance of the known non-coding RNA (ncRNAs) from which the sRNAs were processed (Figure 2C). For example, sRNAs processed from box C/D snoRNAs were enriched in the nucleus (3.3%) and nearly depleted in the cytoplasm (0.08%). In contrast, mitochondrial RNA-derived sRNAs accumulated in the cytoplasm (8.6%) and were depleted in the nucleus (0.63%). Interestingly, most classes of sRNAs exhibited different length distributions in the nuclear and cytoplasmic sRNA libraries (Figure S1), implying that they might be produced via different biogenesis pathways in the nucleus versus the cytoplasm.

**Figure 3:**
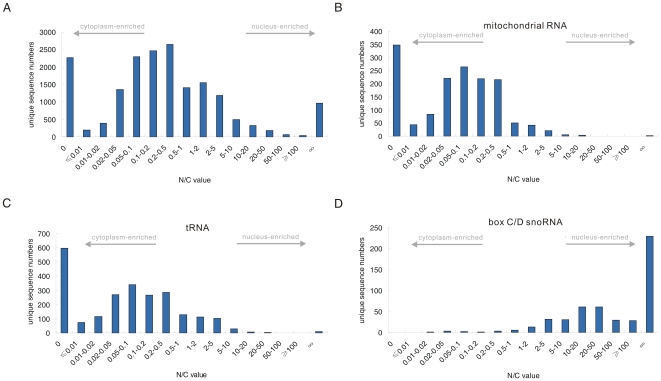
Distribution of N/C values for diverse sRNAs. (*A*, *B*, *C*, *D*) Distribution of N/C values of (*A*) all sRNAs, (*B*) mitochondrial RNA-derived sRNAs, (*C*) tRNA and (*D*) box C/D snoRNA-derived sRNAs.

A total of 17,839 unique sequences presented raw read counts (non-normalized) greater than 10 in at least one library, representing the majority of sRNAs in the 5-8F cell line. We calculated the ratio of nuclear sRNA library counts to cytoplasmic sRNA library counts (N/C) of these unique sequences. The N/C values of the majority of sRNAs (72.4%) ranged from 0.02 to 5 (Figure 3A). Some sRNAs were identified exclusively in the nuclear (5.4%) or cytoplasmic (12.8%) sRNA libraries, but the majority of these were present at a relatively low abundance. The N/C values of the unique sequences could reflect their subcellular distribution. Each of the sRNA class had its own characteristic distribution pattern of N/C value and the sRNAs had a very broad range of N/C values (Table S1, Figure 3A, Figure S2), suggesting a complex subcellular distribution of the sRNAs. Larger N/C values indicate greater enrichment in the nucleus, whereas smaller values indicate greater enrichment in the cytoplasm. As expected, the sRNAs derived from cytoplasmic-localized RNAs, such as mitochondrial RNAs and tRNAs, displayed relatively low N/C values (Figure 3B, 3C, Figure S2) and the sRNAs derived from nuclear-localized RNAs, such as box C/D snoRNAs and rRNA external transcribed spacer (ETS), displayed relative high N/C values (Figure 3D, 5B, Figure S2).

It is important to note that some sRNAs derived from compartment-specific RNA families, such as mitochondrial RNAs and snoRNAs, displayed significantly higher or lower N/C values than other family members (Figure 3B, 3D, Figure S2). For example, the N/C values of a few sRNAs derived from mitochondrial RNAs were larger than 1, much higher than most of the other mitochondrial RNA-derived sRNAs, which presented N/C values lower than 0.1. This suggests that these few sRNAs do not display the same subcellular localization as their precursor or host RNAs.

### Most miRNAs are imported into the nucleus

**Figure 4:**
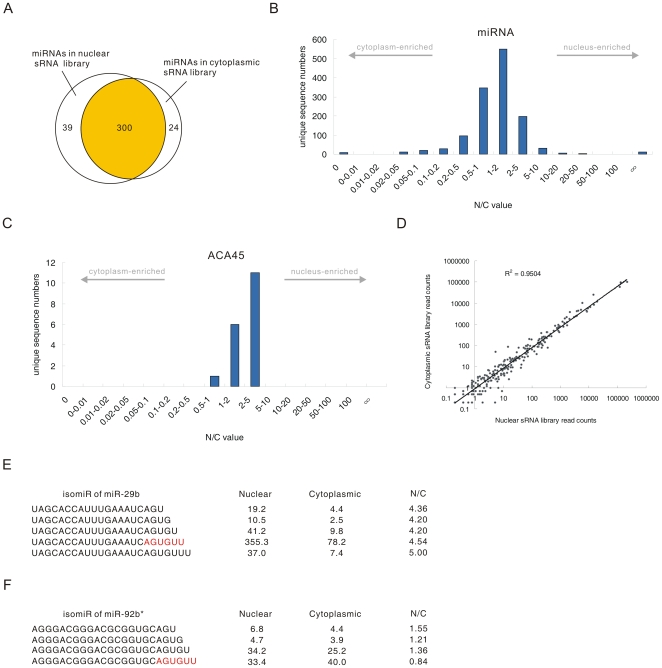
miRNAs in the nuclear and cytoplasmic sRNA libraries. (*A*) Numbers and overlap of miRNAs between the nuclear and cytoplasmic sRNA libraries. (*B*) Distribution of miRNA N/C values. (*C*) Distribution of N/C values for ACA45-derived sRNAs. (*D*) Pearson correlation scatter plot of miRNA levels between the nuclear and cytoplasmic sRNA libraries (R^2^ = 0.9508). (*E*) Different isomiRs of miR-29b displayed similar N/C values. Red letters indicate the nuclear import element of miR-29b reported by Hwang et al. [9]. Nuclear indicates the nuclear sRNA library, and cytoplasmic indicates the cytoplasmic sRNA library. (*F*) N/C values of different isomiRs of miR-92b*.

Additional investigations were carried out to explore the miRNA profiles in further detail. In this study, a total of 339 and 324 miRNAs, corresponding to 361 and 334 distinct miRNA genes, were identified in the nuclear and cytoplasmic sRNA libraries, respectively (Table S2). We identified a large overlap of miRNAs between the two libraries (Figure 4A), and those miRNAs present exclusively in either the nuclear or the cytoplasmic sRNA libraries were generally present at very low abundance. Each miRNA had multiple mature variants, called isomiRs [27]. Although miRNAs have been generally considered to localize and function only in the cytoplasm, most isomiRs with raw read count more than 10 in at least one library (98.5%) were found to present in both the nuclear and cytoplasmic sRNA libraries and microRNAs (Figure 4B) displayed very different N/C value distribution from the sRNAs derived from mitochondrial RNAs (Figure 3B) and box C/D snoRNAs (Figure 3D), indicating that most miRNAs, regardless of their sequence, are imported into the nucleus. Remarkably, the N/C values and length distribution of the sRNAs processed from the ACA45 scaRNA (small cajal body specific RNA) (Figure 4C, Figure S1B) were similar to the length and N/C values of the miRNAs (Figure 4B, Figure S1A), but different from other scaRNA-derived sRNAs (Figure S2A, S1C). This result is consistent with the recent finding that ACA45 can be processed into miRNA-like functional sRNAs [15] and provides support for the import of miRNAs into the nucleus, regardless of their processing pathway.

We employed the read count of the most abundant isomiR to represent the expression level of each miRNA in the two libraries, as previous reports have indicated that the most abundant isomiR is the most useful for identifying differentially expressed miRNAs [27]. A small subset of miRNAs presented different most-abundant isomiRs in the two libraries (Table S3). Since these miRNAs account for only a small portion of the total miRNAs found in the 5-8F cell line and the majority of them were expressed at very low levels (<10 in both libraries), they were not used in further analyses. Therefore, a total of 281 and 266 different mature miRNAs were analyzed from the nuclear and cytoplasmic sRNA libraries, respectively. We found that the expression level of each miRNA in the nuclear miRNA pool correlated with the expression level in the cytoplasmic miRNA pool (R^2^ = 0.9508) (Figure 4D).

A previous report has shown that miR-29b is enriched in the nucleus via a process directed by the 3′ end hexanucleotide AGUGUU [9]. Consistent with this report, our results showed that miR-29b was highly enriched in the nucleus (N/C = 4.54). Several other miRNAs, such as miR-32 (N/C = 6.24), miR-148a (N/C = 4.87) and miR-148b (N/C = 3.72) (Table 2) were also enriched in the nucleus to a similar extent as miR-29b. However, we did not observe conserved 3′ end hexanucleotides or any other conserved elements between the miRNAs with high N/C values, implying that a mechanism other than that directed by hexanucleotides is involved in miRNA import into the nucleus.

**Table 2:** Top 10 miRNAs with the highest N/C values.

microRNA	Sequence of the most abundant isomiR	3′ end hexanucleotide	Nuclear	Cytoplasmic	N/C
hsa-miR-32	UAUUGCACAUUACUAAGUUGCAU	UUGCAU	15.6	2.5	6.24
hsa-miR-148a	UCAGUGCACUACAGAACUUUGU	CUUUGU	41.9	8.6	4.87
hsa-miR-29b	UAGCACCAUUUGAAAUCAGUGUU	AGUGUU	355.3	78.2	4.54
hsa-miR-148b	UCAGUGCAUCACAGAACUUUGU	CUUUGU	145.1	39	3.72
hsa-miR-1	UGGAAUGUAAAGAAGUAUGUAU	AUGUAU	43.4	12.5	3.47
hsa-miR-1285	UCUGGGCAACAAAGUGAGACCU	AGACCU	19	5.9	3.22
hsa-miR-652	AAUGGCGCCACUAGGGUUGUG	GUUGUG	10.7	3.7	2.89
hsa-miR-29c	UAGCACCAUUUGAAAUCGGUUA	CGGUUA	222.7	78.4	2.84
hsa-miR-15b	UAGCAGCACAUCAUGGUUUACA	UUUACA	52.6	19.1	2.75
hsa-miR-135b	UAUGGCUUUUCAUUCCUAUGUGA	AUGUGA	13.5	5.1	2.65

miRNAs are shown only if their most abundant isomiR displayed a raw read count (non-normalized) greater than 10 in at least one library. Nuclear and cytoplasmic indicate the read counts in the nuclear and cytoplasmic sRNA libraries, respectively.

It is worth noting that some miR-29b isomiRs contained shortened or extended versions of the 3′ end motif “AGUGUU”, i.e. “AGU”, “AGUG”, “AGUGU”, and “AGUGUUU”. Interestingly, these isomiRs all presented similar N/C values as the miR-29b containing a complete “AGUGUU” hexanucleotide sequence at its 3′ end (Figure 4E). However, we found that the other miRNAs with these motifs at their 3′ end did not have high N/C value (Table S4), indicating that these motifs could not directed the nuclear enrichment of other miRNAs. We next asked whether the “AGUGUU” motif could direct the nuclear enrichment of other miRNAs. Only one isomiR, an isomiR of miR-92b*, contained the “AGUGUU” sequence at its 3′ end (Figure 4F). However, the nuclear enrichment of this isomiR (N/C = 0.84) was far lower than that of miR-29b. Therefore, “AGUGUU” might only direct the nuclear enrichment of miR-29b in vivo. We further attempted to identify nuclear import elements other than the “AGUGUU” motif by investigating the correlation between specific nucleotide stretches of isomiRs and their N/C values. Our analysis showed that there was no correlation between high N/C value and specific 3′ end nucleotide sequences in isomiRs (Table S5). Therefore, it appears that no nuclear import elements are conserved in the 5′ or 3′ terminal sequences of miRNAs.

### A large number of tRNA 3′ trailers are exported to the cytoplasm

**Figure 5:**
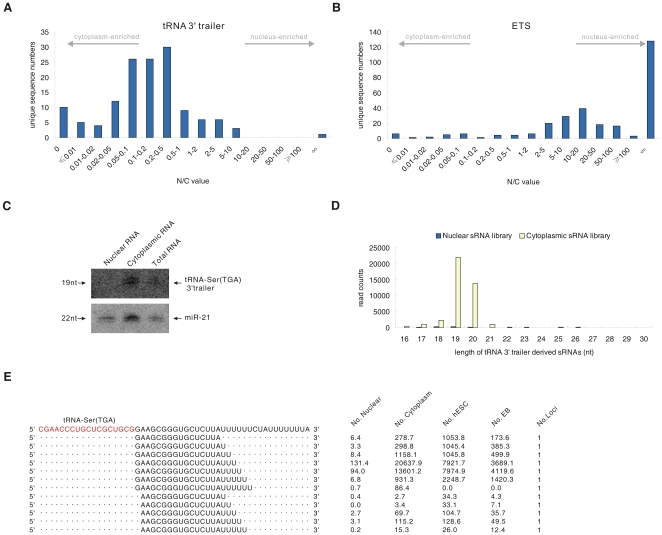
Characteristics of tRNA 3′ trailers. (*A*, *B*) Distribution of N/C values of sRNAs derived from (*A*) tRNA 3′ trailers and (*B*) external transcribed spacers (ETS) of rRNA. (*C*) Northern blot analysis of the tRNA-Ser (TGA) 3′ trailer in nuclear, cytoplasmic and total RNA from the 5-8F cell line. (*D*) Length distribution of sRNAs derived from tRNA 3′ trailers. (*E*) Isotrailers derived from precursors of tRNA-Ser (TGA). The sequence of tRNA-Ser (TGA) is highlighted.

It is well known that pre-tRNAs contain 5′ leader sequences and 3′ trailer sequences that are removed in the nucleus by RNaseP and RNaseZ, respectively [30]. These tRNA 3′ trailers are expected to be degraded in the nuclear compartment soon after being cleaved from pre-tRNAs. In our deep sequencing data, a total of 58 tRNA 3′ trailers with total counts of all isotrailers (various sequences derived from the same 3′ trailer of pre-tRNA) more than 10 in at least one library were detected (Table S6). Unexpectedly, the detected tRNA 3′ trailers displayed relatively small N/C values (Figure 5A) compared with the N/C value of the sRNAs derived from the nuclear specific box C/D snoRNA (Figure 3D) and the rRNA external transcribed spacer (ETS) (Figure 5B), which is also processed in the nucleus. The N/C value distribution of tRNA 3′ trailers is similar to that of sRNA derived from cytoplasmic specific RNAs, such as mitochondrial RNAs (Figure 3B). These findings suggest that tRNA 3′ trailers are exported to the cytoplasm after being processed in the nucleus. The accumulation of one of the most abundant trailer which derived from tRNA-Ser (TGA) precursor in the cytoplasm was further confirmed by the northern blot. A ∼19-nt band was clearly detected by a specific probe in the cytoplasmic RNA fraction (Figure 5C). The presence of different tRNA 3′ trailers with a very broad range of N/C values and abundance also implies that the processing and nuclear export of tRNA 3′ trailers might involve unknown regulatory steps (Table 3, Table S7).

**Table 3:** Five most abundant human tRNA 3′ trailers.

tRNA	Sequence	Cytoplasmic	Nuclear	hESCs	EBs	N/C
tRNA-Ser (TGA)	GAAGCGGGUGCUCUUAUUU	39889.0	264.2	21617.0	10396.7	0.00662
tRNA-Arg (ACG)	GUGUAAGCAGGGUCGUUUU	1176.6	196.0	371.9	165.5	0.16658
tRNA-Ala (CGC)	AGGCGAUCACGUAGAUUUUGUUUA	853.0	203.3	2076.1	534.6	0.23834
tRNA-Thr (CGT)	AGGGUGUGCGUGUUUUUUU	349.3	144.7	172.1	54.7	0.41426
tRNA-Phe (GAA)	GAGAGCGCUCGGUUUUU	316.9	23.7	149.9	32.3	0.07479

We employed the total count of all isotrailers of a given tRNA 3′ trailer to represent its read count. The sequence indicates the most abundant tRNA 3′ trailer sequence from the cytoplasmic sRNA library. Cytoplasmic, nuclear, hESCs and EBs represent the read counts in the cytoplasmic, the nuclear, the human pluripotent embryonic stem cells and the human embryoid bodies sRNA libraries, respectively. Read counts of each sequence were normalized to reads per million (RPM).

Although tRNA 3′ trailers are generally considered to be by-products of tRNA maturation, our analysis demonstrated several interesting features of these RNAs. First, multiple isotrailers from various tRNA 3′ trailers exhibited relatively consistent 5′ ends and heterogeneous 3′ ends (Figure 5E), similar to mature variants of miRNAs [31]. These trailers might result from precise cleavage by RNase Z, generating products with consistent 5′ ends [32], and transcription by polymerase III, which can produce variant 3′ ends [33]. Second, the length of the tRNA 3′ trailers was ∼19-nt (Figure 5D), similar to the lengths of miRNAs and siRNAs. Third, most tRNA 3′ trailers (46/58, 79.3%) terminated with stretches of uridine of various lengths, such as UU, UUU or UUUU, similar to pre-tRNAs [34], [35]. More interestingly, almost all of the tRNA 3′ trailers identified in our two libraries can also be found in pluripotent human embryonic stem cells (hESCs) and embryoid bodies (EBs) sRNA libraries [27] (Table S7), indicating that these tRNA 3′ trailers accumulate in various cell types, including normal human cells. Although the sequences of human tRNA 3′ trailers are not phylogenetically conserved in vertebrates (data not shown), accumulation of diverse tRNA 3′ trailers can also be found in some other vertebrates, including dogs and chickens, and these 3′ trailers share characteristic features with human tRNA 3′ trailers (Table 4, Table 5, Table S7). Together, these data reveal that a large number of tRNA 3′ trailers of ∼19-nt in length are exported out of the nucleus (where they are processed) and accumulate in the cytoplasm, although their biological functions remain unknown.

**Table 4:** Five most abundant chicken tRNA 3′ trailers.

tRNA	Sequence	CE5	CE7	CE9
tRNA-Met (CAT)	AAGGCGGGCAAUGCUUUUC	3021.0	9964.0	4073.1
tRNA-Ile (AAT)	UAAGCGGUAGUCUUUU	500.5	2228.1	261.2
tRNA-Gln (TTG)	GGUGGACAGGGGUCACUUUU	2153.1	1742.6	978.9
tRNA-Tyr (GTA)	GACGGCGAUAUAUUUU	93.3	1193.7	149.5
tRNA-Arg (CCT)	AGCGGAUUGCCCUUUUCUCGCUUUU	67.7	331.0	29.3

We employed the total count of all isotrailers of a given tRNA 3′ trailer to represent its read count. The sequence indicates the most abundant tRNA 3′ trailer sequence from the CE5 sRNA library. CE5, CE7 and CE9 represent the read counts in the 5-day-old chicken embryo, the 7-day-old chicken embryo and the 9-day-old chicken embryo sRNA libraries, respectively. Read counts of each sequence were normalized to reads per million (RPM).

**Table 5:** Five most abundant dog tRNA 3′ trailers.

tRNA	Sequence	Dog lymphocyte
tRNA-Leu (TAG)	ACCCAGGACGGUCUCA	9612.7
tRNA-Ser (TGA)	AAGCGGGUGGCUUCUCUUUU	237.1
tRNA-Arg (CCG)	UGCAGUGGUCGUUUUU	54.3
tRNA-Thr (AGT)	AACCGAGCGUCCCGGCUCUCUU	25.8
tRNA-Val (AAC)	GCAGCGGGCACUGUUGCUUUU	11.1

We employed the total count of all isotrailers of a given tRNA 3′ trailer to represent its read count. The sequence indicates the most abundant tRNA 3′ trailer sequence from the Dog lymphocyte sRNA library. Dog lymphocyte represents read count in the dog lymphocyte sRNA library. Read counts of each sequence were normalized to reads per million (RPM).

### Identification of 26 novel miRNAs from unannotated sRNAs

**Figure 6:**
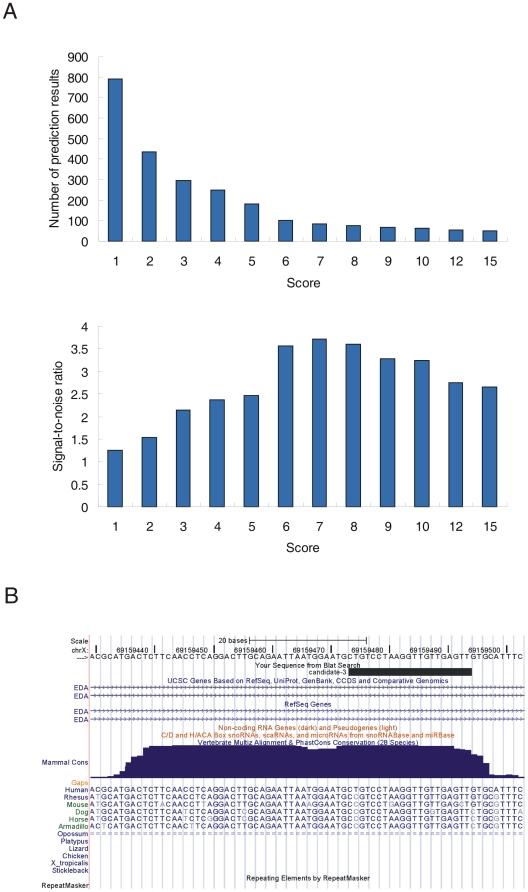
Prediction of novel miRNAs by miRDeep. (*A*) Number of novel miRNA candidates (upper panel) and values of signal-to-noise ratio (lower panel) under different score cutoffs. (*B*) Candidate-3 locates at a conserved genomic region. Shown is screenshot from the UCSC genome browser. The uppermost black bar represents the positions of the presumed mature miRNA. The blue contoured area indicates the level of genomic DNA conservation.

We sought to identify novel miRNAs from the reads mapped to unannotated genomic regions using a recently described miRNA-discovery algorithm, miRDeep, which can identify miRNAs efficiently from deep sequencing data [36], [37]. As sequencing depth affects the prediction accuracy of miRDeep, we used pool reads from our two libraries as well as hESCs and EBs sRNA libraries [27] to predict novel miRNAs. In total, 83 candidates passed the cutoff of 7, a constraint that provided the highest signal-to-noise ratio of the predictions (Figure 6A). After filtering (see Methods for details), 26 candidates were identified and considered to be novel miRNAs (Table 6, Table S7). The majority of the novel miRNAs (21/26, 80.8%) displayed at least one isomiR in the 5-8F cell line, the hESCs and the EBs (Table S7), and nine of the novel miRNAs are expressed in other human tissues or cell lines [36], [38], [39] (Table S8). Remarkably, one of the novel miRNAs (candidate-3) displayed a conservation pattern typical for miRNAs in mammals (Figure 6B). Furthermore, the presumed mature form of this candidate was found in the deep sequencing dataset of sRNAs extracted from Ago proteins [15]. Together, these findings provide further support for the hypothesis that the sRNA candidates are bona fide miRNAs.

**Table 6:** Novel miRNAs identified in this study.

Name	Most abundant sequence	Score	Count*			
			Nuclear	Cytoplasmic	hESCs	EB
candidate-1	UCAGGUGUGGAAACUGAGGCAG	129	73	34	10	7
candidate-2	UGUAGAUACGAGCACCAGCCAC	73.6	40	26	1	0
candidate-3	CUGUCCUAAGGUUGUUGAGUU	53.2	3	5	28	15
candidate-4	UGUCCUCUAGGGCCUGCAGUCU	41.2	14	14	5	6
candidate-5	AAUCUGAGAAGGCGCACAAGGUUU	34.4	4	4	3	5
candidate-6	CAAAAGUGAUCGUGGUUUUUG	27.8	3	1	11	4
candidate-7	AUCAGGGCUUGUGGAAUGGGAAG	24.9	7	1	3	6
candidate-8	AGAAGGGGUGAAAUUUAAACGU	24.7	11	9	3	2
candidate-9	UAAGGGGUGUAUGGCAGAUGCA	22.5	24	8	1	0
candidate-10	ACAGGCGGCUGUAGCAAUGGGGG	18.6	0	0	7	4
candidate-11	UAAAUAGAGUAGGCAAAGGACA	16.3	8	5	10	1
candidate-12	AAUUCCCUUGUAGAUAACCCGG	14.6	1	0	8	5
candidate-13	GGCGACAAAACGAGACCCUGUC	13.3	6	6	0	3
candidate-14	GCUGCACCGGAGACUGGGUAA	12.7	0	9	1	0
candidate-15	UGAGCACCACACAGGCCGGGCGC	10.7	0	0	0	4
candidate-16	AAAAGUAAUCACUGUUUUUGCC	10.4	0	3	3	1
candidate-17	UACGCGCAGACCACAGGAUGUC	10.1	1	1	4	3
candidate-18	CAGCCCGGAUCCCAGCCCACUU	9.9	2	0	3	0
candidate_19	UGAGGGACAGAUGCCAGAAGCA	8.7	3	7	0	1
candidate_20	AGAUAUUUUGAGUGUUUGGAAUUG	8.6	1	1	4	2
candidate_21	UUACACACAACUGAGGAUCAUA	8.6	1	0	0	3
candidate_22	UCUGUAUUCUCCUUUGCCUGCA	8.3	1	0	3	0
candidate_23	AAGCAAUACUGUUACCUGAAAU	8.3	0	1	4	2
candidate_24	UAGCCCCCAGGCUUCACUUGGCG	8.1	0	0	5	0
candidate_25	UUCGGGCUGGCCUGCUGCUCCGG	7.9	0	0	3	1
candidate_26	AGGGCAUAGGAGAGGGUUGAUAU	7.6	5	0	0	0

*The count represents the raw read count of the most abundant sequence of each novel miRNA gene.

## Discussion

**Figure 7:**
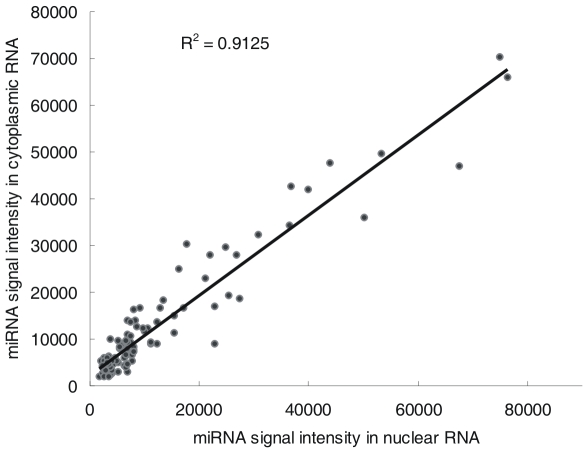
Pearson correlation scatter plot of miRNA expression levels between the nuclear and cytoplasmic sRNAs of 293T cell line (R^2^ = 0.9508).

miRNAs have generally been considered to be cytoplasmic-localized small regulatory RNAs that regulate gene expression predominantly at the post-transcriptional level in the cytoplasm. However, recent studies have reported that a few miRNAs, such as miR-320, miR-373 and miR-29b, localize to or function in the nucleus [9]–[11]. Consistent with these findings, our results revealed that most miRNAs found in the cytoplasm might also localize to the nucleus in the 5-8F cell line. We have not compared the sRNA levels in the transformed cell line and its parental cell line which might have a different pattern, however, we measured the level of expression of miRNAs in other human cells (i.e., 293T cells) using microRNA arrays. Although the miRNA profiling in 293T cells revealed a very different expression pattern from that in 5-8F cells, almost all miRNAs found in the cytoplasm were also detected in the purified nuclear RNA fraction (Table S9). Furthermore, the abundance of most miRNAs in nuclear RNA generally correlated with the abundance in cytoplasmic RNA (Figure 7, Table S9). This indicates that the import of most miRNAs into the nucleus may be a general phenomenon that occurs in a variety of human cells.

Pre-miRNAs are cleaved by Dicer in the cytoplasm to generate a short dsRNA. One strand of this dsRNA is then incorporated into the Ago proteins, while the other is rapidly degraded [40]. Studies have reported that Ago proteins can be imported into the nucleus [18], [19], [21], [22]. Our findings that most miRNAs are imported into the nucleus and that the nuclear and cytoplasmic abundances of miRNAs are correlated imply that miRNAs might be co-imported with the Ago proteins into the nucleus. This hypothesis is further supported by evidence that the majority of isomiRs processed from ACA45 are incorporated into Ago protein complexes [15] and that they are also imported into the nucleus, as we have shown in this study. Although Ago proteins might be imported into the nucleus while complexed with miRNAs, we found that the nuclear entry ability of different miRNAs was highly diverse based on their N/C values. (Table S2). It is likely that the nuclear import of Ago proteins is affected by other regulatory factors, such as the sequence of the associated mature miRNAs.

The fact that most miRNAs are imported into the nucleus suggests that numerous, instead of a few, miRNAs might play regulatory roles in the nucleus although the nuclear targets of miRNAs are largely unknown. To date, only two miRNAs, miR-320a and miR-373, have been demonstrated to regulate gene expression in the nucleus through targeting the promoter regions of protein-coding genes [10] [11]. However, many protein-coding genes were found to contain sites of near-perfect complementarity to many mature miRNAs in their promoter regions [10]. Moreover, studies of miRNA target sequences interacting with the Ago proteins identified a substantial number of sequences derived from introns, which are predominantly present in the nucleus [41]. In addition, MALAT-1, a nuclear-localized ncRNA [42], was also found to be associated with the Ago2 protein [18]. Together, these data suggest that miRNAs have numerous potential targets in the nucleus. The systematic identification of the nuclear targets of miRNAs would provide further insight into the phenotypic consequences associated with certain miRNAs and the roles that miRNA play in the nucleus.

In previous cloning efforts, sRNAs with exact matches to various ncRNAs were frequently dismissed as nonfunctional degradation products. However, recent studies have shown that many known ncRNAs, such as snoRNAs, vRNAs (vault RNA), tRNAs, etc… [15], [43]–[45] can be further processed into small regulatory RNAs, termed ncRNA-associated sRNAs (nasRNAs) [46]. Our analysis provide evidence to support that some sRNAs may not simply be non-functional degradation products: (1) some sRNAs accumulate to a very high abundance (Table S1), (2) each class of sRNAs displayed different characteristic length distribution patterns in nuclear and cytoplasmic sRNA libraries. (Figure S1) and (3) some sRNAs localized differently than their precursors, implying that they traffic between compartments of the cell.

Previous reports have indicated that the transcription of some ncRNAs, such as miRNAs [47] and snoRNAs [48], could be driven by the tRNA promoter. Our results revealed that tRNA 3′ trailers, sequences that have traditionally been expected to be degraded after being cleaved from tRNA precursors, might also act as functional sRNAs driven by the tRNA promoter. Several lines of evidence support the speculation that some tRNA 3′ trailers have biological functions. First, some tRNA 3′ trailers were exported to the cytoplasm after production in the nucleus and accumulated to a high abundance in the cytoplasm. Second, tRNA 3′ trailers that accumulated in 5-8F cells also accumulated in normal human cells (Table S7). Third, tRNA 3′ trailers also accumulated in the cells of other vertebrates, including dogs and chickens (Table S7), suggesting a conserved biogenesis mechanism and a potential function of tRNA 3′ trailers in vertebrates. The exact transport process of the tRNA trailers in cells is still unknown, but the nuclear export of tRNA 3′ trailers might be mediated by the La protein which binds to tRNA 3′ trailers during processing and modification of pre-tRNAs [49], since the RNA recognition motif (RRM) of the La protein displays nuclear export activity [50].

Advances in high-throughput next-generation sequencing technology have greatly transformed the transcriptomic research landscape. Using this technology, we identified an unexpectedly complex subcellular distribution of miRNAs and tRNA 3′ trailers and also identified numerous unannotated sRNAs, including novel miRNA-producing regions of the human genome. As recently demonstrated, there remain many unknown sRNAs in human cells [38], [46], and the characterization of the subcellular distributions of human sRNAs might provide hints toward the identification of novel sRNA structures and their functions.

## Materials and Methods

### Preparation of nuclear, cytoplasmic and total RNA

Human Nasopharyngeal carcinoma cell lines 5-8F was purchased from the Cancer Research Institute of Sun Yat-sen University (Guangzhou, China). Cells was maintained at 37°C in DMEM containing 10% FBS. Cells were collected by centrifugation and washed with PBS (pH 7.4). Total RNA was isolated from cells using the method of guanidine thiocyanate/phenol – chloroform [51]. Nuclear and cytoplasmic fractions were isolated according to the method of Greenberg et al. [52] and Qiagen (Valencia, CA) RNeasy Minikit protocol with some modifications. Briefly, the cell pellet was resuspended in 20 pellet volumes of RLN buffer (50 mM Tris-HCl, pH 7.4, 0.14 M NaCl, 1.5 mM MgCl_2_, 0.5% IGEPAL CA-630 (Sigma), 1 U/ul RNase Inhibitor (TaKaRa), 1 mM DTT) and incubated for 5 min on ice. The nuclei were collected by centrifugation at 300 g for 3 min at 4°C. The supernatant was used to isolate cytoplasmic RNA using the same method that used to isolate total RNA. Nuclei pellet was resuspended in 20 pellet volumes of RSB buffer (0.25 M sucrose, 10 mM Tris-HCl, pH 7.4, 10 mM NaCl, 3 mM MgCl_2_, 1 mM DTT), then nuclei were collected by ultracentrifugation through a sucrose cushion. Nuclear RNA was isolated from sucrose gradient centrifugation purified nuclei using the same method used to isolate cytoplasmic RNA. The integrity of the RNA was assessed using denaturing agarose gel electrophoresis. The nuclear and cytoplasmic RNAs were run on a 10% denaturing polyacryamide gels to assess whether the nuclear and cytoplasmic RNAs were successfully separated.

### sRNA library preparation and sequencing

sRNA library preparation and Solexa sequencing was performed by Beijing Genomics Institute (BGI) at ShenZhen according to the manufacturer's instructions. Briefly, sRNAs ranging from 18 to 30-nt were gel-purified and ligated to the 3′ adaptor (5′-pUCGUAUGCCGUCUUCUGCUUGidT-3′; p, phosphate; idT, inverted deoxythymidine) and 5′ adaptor (5′-GUUCAGAGUUCUACAGUCCGACGAUC-3′). Ligation products were gel-purified, reverse transcribed, and amplified using Illumina's sRNA primer set (5′-CAAGCAGAAGACG GCATACGA-3′; 5′-AATGATACGGCGACCACCGA-3′). Samples were sequenced on an Illumina 1G Genome Analyzer.

### Bioinformatic analysis of sRNA libraries

The 3′ adaptor sequences were removed from the Illumina-generated reads at BGI Shenzhen using a dynamic programming algorithm that required at least 5-nt overlap between 35-nt reads and the 3′ adaptor sequence. After removing the reads without the adaptor sequences, poly-A reads and 5′ adaptor contaminants, the remaining 16- to 30-nt reads were mapped to the UCSC hg18 assembly of the human genome [53] using bowtie [54] with the following options: –f –n 0 –a. All reads that could be aligned to the human genome were moved to the “mapped” data set. The remaining reads constituted the “unmapped” data set. We next moved the reads from the “unmapped” data set that could be aligned to rRNA genes downloaded from the NCBI (Accession number: U13369), the exon-exon junction of the tRNA and mRNA, or the tRNA 3′ end that contained the post-transcriptionally-added CCA into the “mapped” data set. Each sequence in “mapped” data set was annotated by simply aligning to known RNA sequences which were downloaded from Genebank (http://www.ncbi.nih.gov/Genbank/index.html, rRNA), snoRNA-LBME-db http://wwwsnorna.biotoul.fr/index.php, snoRNA), UCSC (http://genome.ucsc.edu, various RNAs and repeat sequences), miRBase (http://microrna.sanger.ac.uk/sequences/index.shtml, release version 12.0, miRNA). The annotation order was miRNA, mitochondrial RNA, rRNA, internal transcribed spacer (ITS) of rRNA, external transcribed spacer (ETS) of rRNA, Box H/ACA snoRNA, Box C/D snoRNA, scaRNA, tRNA, snRNA, RNaseP, SRP RNA, Xist RNA, 7SK RNA, H19 RNA, vRNA, hY RNA, RNaseMRP, mRNA and 3′ trailer sequence of tRNA. Considering that some reads could be aligned to multiple kinds of RNA, we removed them from the “mapped” data set when they were first annotated to one kind of RNA. For instance, if some reads were annotated as miRNA, they were removed from the “mapped” data set before the remnant reads were aligned to mitochondrial RNA. We found that many sequences in the “unmapped” data set aligned with the known tRNA sequences with a single nucleotide mismatch, which might be caused by extensive modification of mature tRNA sequences. These sequences were moved to the “mapped” data set and annotated as tRNA-derived sRNAs. The imperfect mapping to the human genome of many reads in the “unmapped” data set might be caused by 3′ untemplated nucleotides [55]–[58]. We removed the 3′ untemplated nucleotides from reads if they possessed an exact match to the human genome starting at the 5′ end and maintained a contiguous match of 17 or more nucleotides. These trimmed reads were then moved to the “mapped” data set. The remnant sequences in the “unmapped” data set were discarded. All reads in the “mapped” data set were compiled into a set of unique sequences, with the number of reads for each sequence reflecting relative abundance [59]. The read count of each unique sequence was normalized to reads per million (RPM), according to the total read count of the “mapped” data set.

### Analysis of tRNA 3′ trailers and the sRNA deep sequencing data sets used in this study

40 nucleotides immediately downstream of each tRNA gene of human, chicken and dog were downloaded from UCSC (http://genome.ucsc.edu), and sRNA sequences from several human, chicken and dog sRNA deep sequencing data sets were mapped to these downloaded sequences. sRNAs which perfectly matched from the first or second nucleotide of any downloaded sequence were considered to be tRNA 3′trailers. Additional sRNA deep sequencing data sets used in this study included: pluripotent human embryonic stem cells and embryoid bodies sRNA data set (ftp://ftp03.bcgsc.ca/public/hESC_miRNA/) [27]; Hela and dog lymphocytes sRNA data sets (GEO accession number GSE10825) [36]; chicken sRNA libraries from embryonic days 5, 7 and 9 (GEO accession number GSE10686) [28]; human THP-1 sRNA data sets (DNA Database of Japan,AIAAA0000001–AIAAT0000001) [38]; human serous ovarian cancer tissue, human clear cell ovarian cancer tissue, primary cultures of normal human ovarian surface epithelium and human endometrioid ovarian sRNA data sets (GEO accession number GSE15190) [39]; sRNAs associated with human Ago proteins (GEO accession number GSE13370) [15].

### Novel miRNA prediction

Novel miRNAs were predicted by a recently described algorithm miRDeep [36]. Prediction was performed according to the manual of miRDeep. The data set we used to predicted miRNA comprised pooled reads from nuclear, cytoplasmic, hESCs and EBs sRNA libraries [27]. We discarded the following miRNA candidates: (1) the mature form of candidates had more than 5 loci in the genome or overlap with known RNAs; (2) candidates overlapped with the minus strand of known miRNAs; (3) the length of the mature form of candidates shorter than 19-nt or longer than 25-nt. The signal-to-noise ratio of the prediction was calculated according to the manual of miRDeep. RNA secondary structures were predicted using RNAfold [60].

### Northern blot analysis

Northern blot was performed as described [61] with some modifications. Briefly, 25 micrograms of nuclear, cytoplasmic and total RNAs were run on denaturing 15% polyacrylamide gel, and then electrophoretically transferred to Hybond-N+ membranes (Amersham, GE Life Sciences) using the semidry blotting apparatus (BioRad), followed by UV light irradiation for 4min and baked at 80°C for 50min. DNA oligonucleotides complementary to different ncRNA sequences were synthesized (Sangon, Shanghai). The 5′ ends of the DNA probes were labeled with [γ-^32^P]ATP (Yahui Co.) using T4 polynucleotide kinase (TaKaRa). The membranes were prehybridized for at least 1h in hybridization buffer (5× SSC, 20mM NaH_2_PO_4_ pH 7.2, 7% SDS, 2× Denhardt's Solution) and then were hybridized overnight at 42°C. After being washed three times with 2× SSPE/0.1% SDS at room temperature, the membranes were exposed to a phosphor screen and visualized by Typhoon 8600 variable mode imager (Amersham Biosciences). Probes for northern blot analysis were listed in Table S10. miRNA array was performed as previously described [62]. Membranes were also exposed to a phosphor storage screen, and visualized by Typhoon 8600 variable mode imager. Hybridization signals were quantified using Image Quant software (Molecular Dynamics).

## Supporting Information

Figure S1 Length distribution of diverse sRNAs. Length distribution of (A) miRNAs, (N) unknown sRNAs and sRNA derived from (B) ACA45 scaRNA, (C) scaRNAs (without ACA45), (D) box H/ACA snoRNAs, (E) box C/D snoRNAs, (F) rRNAs (G) rRNA external transcribed spacer (ETS), (H) rRNA internal transcribed spacer (ITS), (I) tRNAs, (J) mitochondrial RNAs, (K) snRNAs, (L) Y RNAs, (M) SRP RNAs, (O) genomic repeats and (P) mRNAs in nuclear and cytoplasmic sRNA libraries. sRNA classes are shown only if they displayed total abundance greater than 1000 in at least one library. Read counts of each sequence were normalized to reads per million (RPM).
(0.81 MB TIF)

Figure S2 Distribution of N/C values for diverse sRNAs. Distribution of N/C values for (I) unknown sRNAs, sRNAs derived from (A) scaRNA, (B) box H/ACA snoRNAs, (C) mRNAs, (D) rRNAs, (E) rRNA internal transcribed spacer (ITS), (F) snRNAs, (G) Y RNAs, (H) SRP RNAs and (J) genomic repeats.
(0.86 MB TIF)

Table S1 The N/C values of diverse sRNAs. sRNAs with raw read counts (non-normalized) more than 10 in at least one library were listed. N/C indicates the ratio of nuclear sRNA library count to cytoplasmic sRNA library count of each unique sequence. Nuclear and cytoplasmic indicate the read counts in the nuclear and cytoplasmic sRNA libraries, respectively. Read count of each sRNA were normalized to reads per million (RPM).
(2.43 MB XLS)

Table S2 Known miRNAs in miRBase release 12.0. In the column MirBase, “5p” and “3p” represent miRNA precursor which is annotated to have mature form locate in 5′ arm and 3′ arm respectively by miRBase, and “*” represents miRNA precursor which is annotated to have miRNA* by miRBase.
(3.08 MB HTML)

Table S3 miRNAs that present different most abundant isomiR in nuclear and cytoplasmic sRNA libraries. Read counts of each sequence were normalized to reads per million (RPM).
(0.03 MB XLS)

Table S4 IsomiRs which had shorten or extended version of “AGUGUU” or various 3′ end hexanucleotides of miR-29b isomiRs at their 3′ end. Red letters represent shorten or extended version of “AGUGUU”. Blue letters represent 3′ end hexanucleotides of various miR-29b isomiRs except “AGUGUU”. The isomiRs are shown only if it displayed a raw read count (non-normalized) more than 10 in at least one library.
(0.03 MB XLS)

Table S5 The N/C values of all isomiRs. The isomiRs are shown only if it displayed a raw read count (non-normalized) more than 10 in at least one library.
(0.26 MB XLS)

Table S6 A large number of tRNA 3′ trailers are accumulated in different cells of human and diverse vertebrates. We employed the total count of all isotrailers of a given tRNA 3′ trailer to represent its read count. tRNA 3′ trailers of human and chicken with raw read counts more than 10 in at least one library were listed. The sequence indicates the most abundant tRNA 3′trailer sequence from cytoplasmic sRNA library in human, CE5 sRNA library in chicken. Cytoplasmic, nuclear, hESCs, EBs, CE5, CE7, CE9 and dog lymphocyte represent the read counts in cytoplasmic, nuclear, human pluripotent human embryonic stem cells, human embryoid bodies, 5-day-old chicken embryo, 7-day-old chicken embryo, 9-day-old chicken embryo and dog lymphocyte sRNA libraries, respectively. C/N indicates the ratio of cytoplasmic sRNA library count to nuclear sRNA library count of each unique sequence. Read counts of each sequence were normalized to reads per million (RPM).
(0.03 MB XLS)

Table S7 Novel miRNAs identified in this study.
(0.14 MB HTML)

Table S8 Novel miRNAs in other tissues or cell lines. “√” indicates that at least one isomiR of novel miRNA gene presented in corresponding sRNA library. Osc, Occ, Opc and Oec represent sRNAs from serous ovarian cancer tissue, clear cell ovarian cancer tissue, primary cultures of normal human ovarian surface epithelium (HOSE) and endometrioid ovarian cancer tissue, respectively.
(0.04 MB DOC)

Table S9 The expression level of all microRNAs that detected in the nuclear and cytoplasmic 18–30nt sRNA of 293T cell line.
(0.03 MB XLS)

Table S10 Probes for Northern blot analysis. All probes were synthesized and purified by Sangon Co. (Shanghai, China).
(0.03 MB DOC)
