# Supplementary figures and images for: Deep Sequencing of Human Nuclear and Cytoplasmic Small RNAs Reveals an Unexpectedly Complex Subcellular Distribution of miRNAs and tRNA 3′ Trailers
Source: PLoS One. 2010 May 14;5(5):e10563. doi: 10.1371/journal.pone.0010563 (PMC2871053; doi:10.1371/journal.pone.0010563)

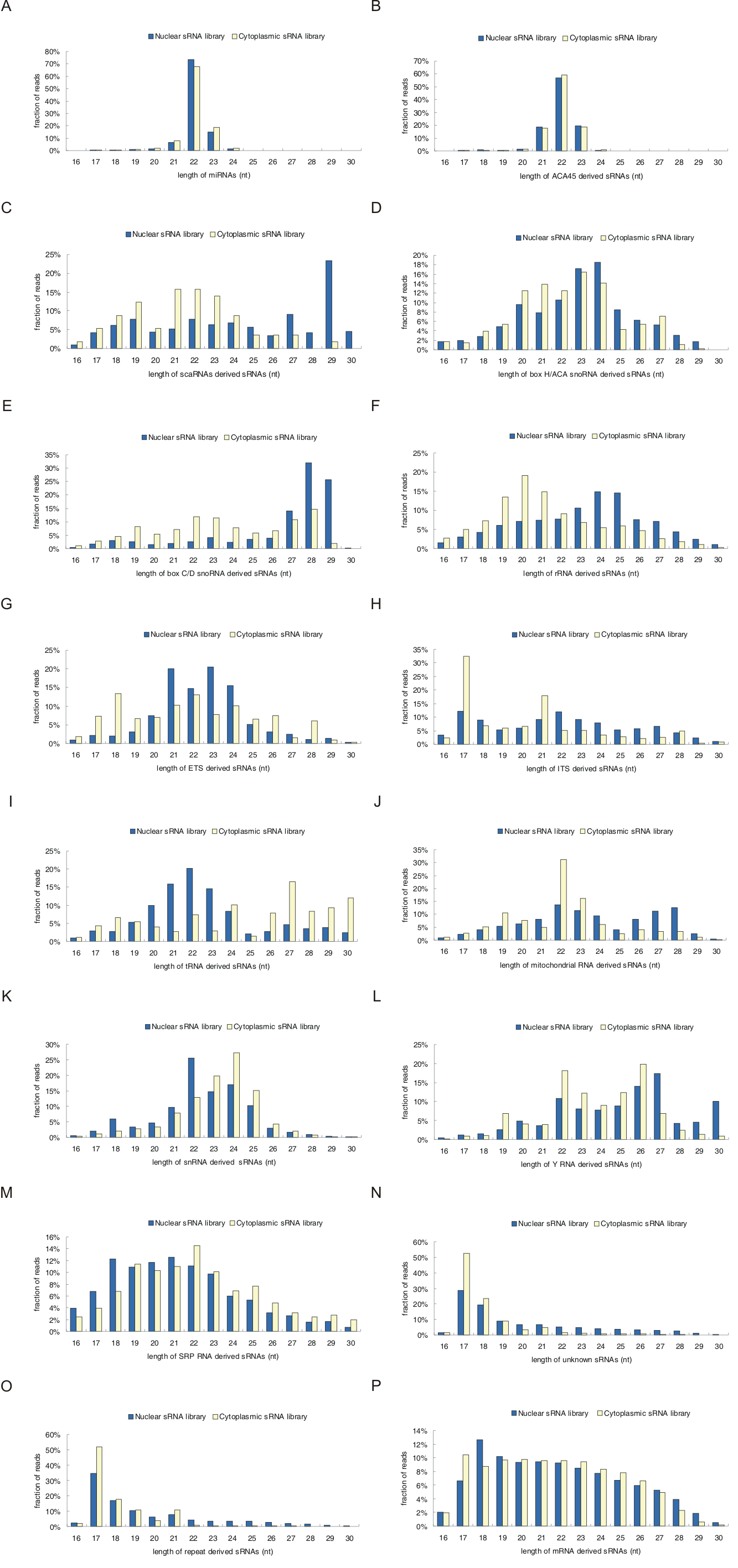

Supplement: Figure S1 — Length distribution of diverse sRNAs. Length distribution of (A) miRNAs, (N) unknown sRNAs and sRNA derived from (B) ACA45 scaRNA, (C) scaRNAs (without ACA45), (D) box H/ACA snoRNAs, (E) box C/D snoRNAs, (F) rRNAs (G) rRNA external transcribed spacer (ETS), (H) rRNA internal transcribed spacer (ITS), (I) tRNAs, (J) mitochondrial RNAs, (K) snRNAs, (L) Y RNAs, (M) SRP RNAs, (O) genomic repeats and (P) mRNAs in nuclear and cytoplasmic sRNA libraries. sRNA classes are shown only if they displayed total abundance greater than 1000 in at least one library. Read counts of each sequence were normalized to reads per million (RPM). (0.81 MB TIF) [file pone.0010563.s001.tif]

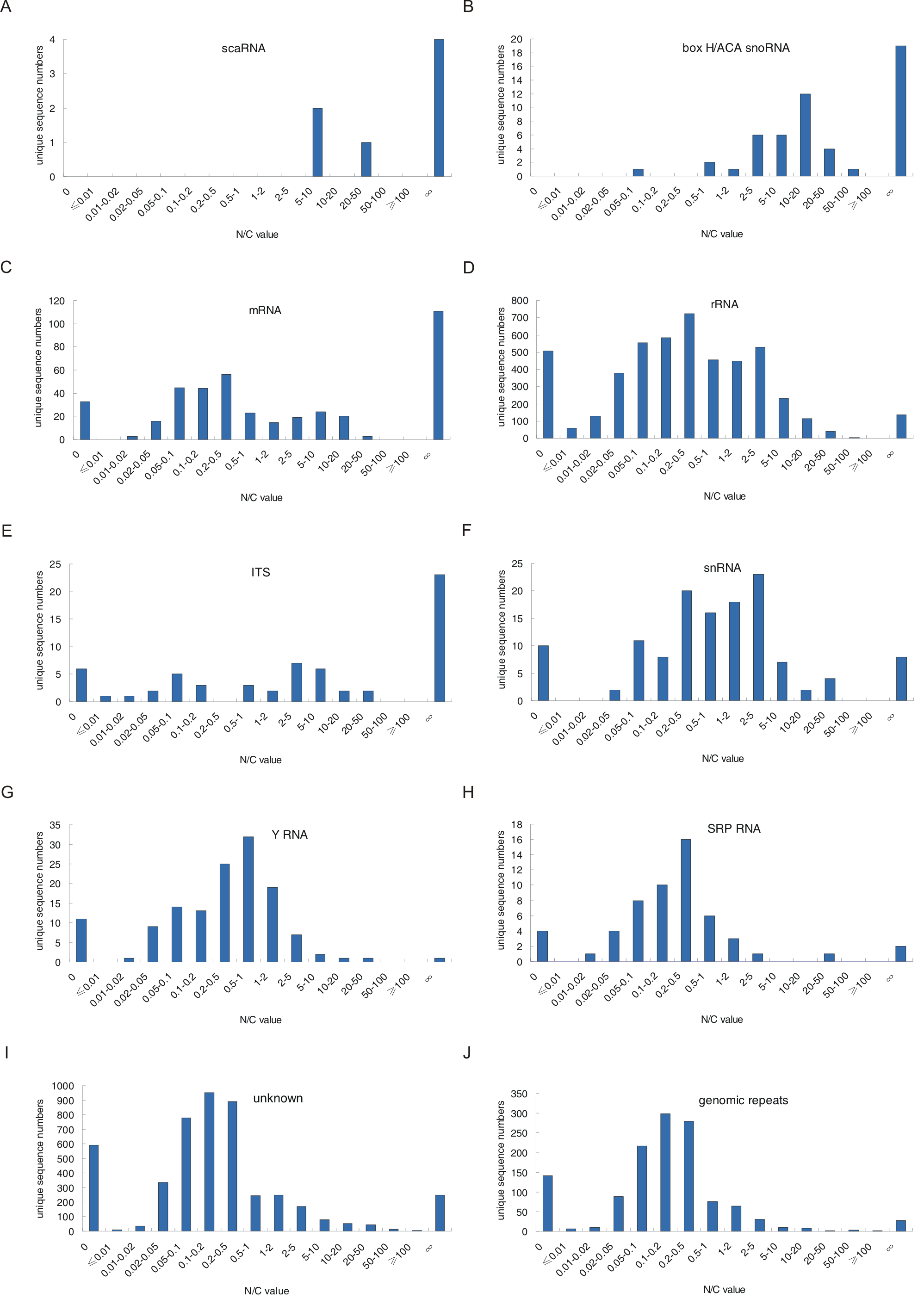

Supplement: Figure S2 — Distribution of N/C values for diverse sRNAs. Distribution of N/C values for (I) unknown sRNAs, sRNAs derived from (A) scaRNA, (B) box H/ACA snoRNAs, (C) mRNAs, (D) rRNAs, (E) rRNA internal transcribed spacer (ITS), (F) snRNAs, (G) Y RNAs, (H) SRP RNAs and (J) genomic repeats. (0.86 MB TIF) [file pone.0010563.s002.tif]
