# Supplementary material for: Deep Sequencing of Human Nuclear and Cytoplasmic Small RNAs Reveals an Unexpectedly Complex Subcellular Distribution of miRNAs and tRNA 3′ Trailers
Source: PLoS One. 2010 May 14;5(5):e10563. doi: 10.1371/journal.pone.0010563 (PMC2871053; doi:10.1371/journal.pone.0010563)
Supplement: Table S2 — Known miRNAs in miRBase release 12.0. In the column MirBase, “5p” and “3p” represent miRNA precursor which is annotated to have mature form locate in 5′ arm and 3′ arm respectively by miRBase, and “*” represents miRNA precursor which is annotated to have miRNA* by miRBase. (3.08 MB HTML) [file pone.0010563.s004.html]

  
  
If this is the only text that appears, enable javascript in your browser.
