# Supplementary material for: Deep Sequencing of Human Nuclear and Cytoplasmic Small RNAs Reveals an Unexpectedly Complex Subcellular Distribution of miRNAs and tRNA 3′ Trailers
Source: PLoS One. 2010 May 14;5(5):e10563. doi: 10.1371/journal.pone.0010563 (PMC2871053; doi:10.1371/journal.pone.0010563)
Supplement: Table S8 — Novel miRNAs in other tissues or cell lines. “√” indicates that at least one isomiR of novel miRNA gene presented in corresponding sRNA library. Osc, Occ, Opc and Oec represent sRNAs from serous ovarian cancer tissue, clear cell ovarian cancer tissue, primary cultures of normal human ovarian surface epithelium (HOSE) and endometrioid ovarian cancer tissue, respectively. (0.04 MB DOC) [file pone.0010563.s010.doc]

**Table S8. Novel miRNAs in other tissues or cell lines.**

|  | THP-1 | Hela | Opc | Oec | Occ | Osc |
| --- | --- | --- | --- | --- | --- | --- |
| candidate-1 |  | √ |  |  |  |  |
| candidate-3 |  |  | √ | √ | √ | √ |
| candidate-4 |  |  | √ | √ | √ | √ |
| candidate-7 |  | √ |  |  |  |  |
| candidate-14 | √ |  |  |  |  |  |
| candidate-18 | √ |  |  |  |  |  |
| candidate_19 |  |  | √ |  |  | √ |
| candidate_21 |  |  | √ | √ | √ | √ |

“√” indicates that at least one isomiR of novel miRNA gene presented in corresponding sRNA library. Osc, Occ, Opc and Oec represent sRNAs from serous ovarian cancer tissue, clear cell ovarian cancer tissue, primary cultures of normal human ovarian surface epithelium (HOSE) and endometrioid ovarian cancer tissue respectively.
