# Supplementary material for: Deep Sequencing of Human Nuclear and Cytoplasmic Small RNAs Reveals an Unexpectedly Complex Subcellular Distribution of miRNAs and tRNA 3′ Trailers
Source: PLoS One. 2010 May 14;5(5):e10563. doi: 10.1371/journal.pone.0010563 (PMC2871053; doi:10.1371/journal.pone.0010563)
Supplement: Table S10 — Probes for Northern blot analysis. All probes were synthesized and purified by Sangon Co. (Shanghai, China). (0.03 MB DOC) [file pone.0010563.s012.doc]

**Table S10. Probes for Northern blot analysis.**

| Probe | Probe sequences (5′ to 3′) |
| --- | --- |
| U78 | TTACCTTTGTCTACATGCTC |
| mitochondrial tRNA-Val | TGGTCAGAGCGGTCAAGTTA |
| tRNA-Lys(TTT) | ACCGACTGAGCTATCCGGGC |
| tRNA-His(GTG) | GCCGTGACTCGGATTCGAACC |
| tRNA-Ser(TGA) 3’ trailer | AAATAAGAGCACCCGCTTC |
| mitochondrial tRNA-Gln | ACCTATCACACCCCATCCTA |
| miR-21 | TCAACATCAGTCTGATAAGCTA |

All probes were synthesized and purified by Sangon Co. (Shanghai, China).
